# The polycyclic aromatic hydrocarbon degradation potential of Gulf of Mexico native coastal microbial communities after the Deepwater Horizon oil spill

**DOI:** 10.3389/fmicb.2014.00205

**Published:** 2014-05-09

**Authors:** Anthony D. Kappell, Yin Wei, Ryan J. Newton, Joy D. Van Nostrand, Jizhong Zhou, Sandra L. McLellan, Krassimira R. Hristova

**Affiliations:** ^1^Department of Biological Sciences, Marquette UniversityMilwaukee, WI, USA; ^2^School of Freshwater Sciences, Great Lakes WATER Institute, University of Wisconsin-MilwaukeeMilwaukee, WI, USA; ^3^Department of Microbiology and Plant Biology, Institute for Environmental Genomics, University of OklahomaNorman, OK, USA

**Keywords:** Deepwater Horizon, oil spill, microbial community composition, high-throughput sequencing, functional gene arrays, polycyclic aromatic hydrocarbons (PAH), biodegradation, mesocosms

## Abstract

The Deepwater Horizon (DWH) blowout resulted in oil transport, including polycyclic aromatic hydrocarbons (PAHs) to the Gulf of Mexico shoreline. The microbial communities of these shorelines are thought to be responsible for the intrinsic degradation of PAHs. To investigate the Gulf Coast beach microbial community response to hydrocarbon exposure, we examined the functional gene diversity, bacterial community composition, and PAH degradation capacity of a heavily oiled and non-oiled beach following the oil exposure. With a non-expression functional gene microarray targeting 539 gene families, we detected 28,748 coding sequences. Of these sequences, 10% were uniquely associated with the severely oil-contaminated beach and 6.0% with the non-oiled beach. There was little variation in the functional genes detected between the two beaches; however the relative abundance of functional genes involved in oil degradation pathways, including polycyclic aromatic hydrocarbons (PAHs), were greater in the oiled beach. The microbial PAH degradation potentials of both beaches, were tested in mesocosms. Mesocosms were constructed in glass columns using sands with native microbial communities, circulated with artificial sea water and challenged with a mixture of PAHs. The low-molecular weight PAHs, fluorene and naphthalene, showed rapid depletion in all mesocosms while the high-molecular weight benzo[α]pyrene was not degraded by either microbial community. Both the heavily oiled and the non-impacted coastal communities showed little variation in their biodegradation ability for low molecular weight PAHs. Massively-parallel sequencing of 16S rRNA genes from mesocosm DNA showed that known PAH degraders and genera frequently associated with oil hydrocarbon degradation represented a major portion of the bacterial community. The observed similar response by microbial communities from beaches with a different recent history of oil exposure suggests that Gulf Coast beach communities are primed for PAH degradation.

## Introduction

The destruction of the Deepwater Horizon (DWH) oil rig resulted in the discharge of approximately 4.9 million barrels of light crude oil into Gulf of Mexico marine environments from April 20, 2010 to July 15, 2010, making it the second worst oil spill in US history (Lehr et al., 2010). During the cleanup effort, crude oil along with added dispersants formed more than a 35 km long plume in the Gulf of Mexico that significantly impacted coastal ecosystems, including native microbial community composition (Camilli et al., 2010) with unknown ecological consequences. Significant amounts of oil washed ashore on marshes and beaches in the Gulf of Mexico over a 2- to 3-month period and were subsequently buried underneath layers of sand (Allan et al., 2012), bringing enormous amounts of allochthonous carbon to the beach ecosystems. An estimated 1.7 × 10^11^ g of C(1)-C(5) hydrocarbons including 2.1 × 10^10^ g of PAHs were released to the water column during the spill (Reddy et al., 2012). The low molecular weight PAH, naphthalene was the dominant PAH in the crude oil followed by the low molecular weight PAHs, phenanthrene and fluorene, while the high-molecular weight PAH, chrysene and other PAHs were minor components (Zhanfei et al., 2012). PAHs, particularly high molecular weight compounds, are one of the major contaminant classes of concern in oil spills because many are toxic and/or carcinogenic to humans and wildlife and are often recalcitrant to degradation in environmental media.

Coastal shores protect inland areas from disturbances like hurricanes by buffering wind and wave energy. Additionally, they provide refuge and nesting ground for many species of marine and land animals. Sandy beaches also serve as the primary location of physical interaction between humans and the marine environment with significant impacts upon human health and local and state economies (USEPA, 2004). Permeable sandy sediments cover large areas of the seafloor in the Gulf of Mexico, including beaches (Huettel and Rusch, 2000). Microbial biofilms cover the sand particles in these coastal ecosystems, and these biofilms harbor phylogenetically and functionally diverse bacteria whose abundances exceed that of the overlying seawater by orders of magnitude (Koster et al., 2005; Hunter et al., 2006; Karnachuk et al., 2006). The permeable sand sediments facilitate porewater exchange, transport of nutrients and removal of waste products for a very active microbial metabolism (Huettel and Rusch, 2000; Rusch et al., 2003).

Sand microbial communities are known to play a major role in the cycling of nutrients in these coastal ecosystems (Hunter et al., 2006; Mills et al., 2008; Huettel et al., 2014), and are likely among the earliest responders to anthropogenic pollution. Microbial communities in the Gulf of Mexico are also thought to be responsible for the intrinsic bioremediation of the crude oil released by the DWH oil spill (Atlas and Hazen, 2011; Chakraborty et al., 2012; Lu et al., 2012). Results from several research groups indicated that hundreds of bacterial taxa can decompose a variety of oil hydrocarbons and rapidly proliferate in the presence of oil in the deep water column (Hazen et al., 2010; Atlas and Hazen, 2011; Lu et al., 2012; Redmond and Valentine, 2012; Valentine et al., 2012; Kimes et al., 2013) but their relative abundance changes as the chemical composition of the oil is modified by the microbial community (Dubinsky et al., 2013). For example, the relative importance of taxa such as uncultivated *Oceanospirillales*, *Pseudomonas*, *Colwellia*, *Cycloclasticus*, *Pseudoalteromonas*, and *Thalassomonas* was controlled by changes in hydrocarbon supply in the water column (Dubinsky et al., 2013). The DWH oil spill dramatically altered not only microbial community composition but also the functional gene structure in the deep sea (Lu et al., 2012). A variety of metabolic genes involved in both aerobic and anaerobic hydrocarbon degradation were highly enriched in the plume compared with outside the plume (Lu et al., 2012). Fewer studies have focused on the impact from increased oil hydrocarbons on the terrestrial microbial communities such as those found in beach sand (Kostka et al., 2011; Newton et al., 2013) and coastal salt marshes (Beazley et al., 2012). In addition, complex hydrocarbon deposition could have major consequences in coastal environments for the entire bacterial community rather than just for those microorganisms capable of using the introduced carbon mixture. Depletion of essential nutrients, such as nitrogen and phosphorus during the hydrocarbon degradation could significantly shift microbial community composition and function.

In this study, we characterized changes in the functional gene composition and abundance of native microbial communities that accompany oil contamination in Gulf beach sands. We also investigated the PAH degradation potential of beach sand microbial communities. To set up comparative analyses, beach sand was collected from a beach in Orange Beach, AL that was severely impacted by oil contamination following the DWH spill and a beach on St. George Island, FL where negligible contamination was detected in the months following the blowout. This allows us to make pre- and post-oil spill comparisons between the communities. Newton et al. (2013) demonstrated the increased abundance of known-oil degraders within the Orange Beach in the month of June suggesting an accompanying detectable change in the functional gene structure of the community. We investigated the beach sand microbial community functional gene response to hydrocarbon exposure using a functional gene microarray (GeoChip 4.2), targeting 539 gene families. Given that the Gulf of Mexico has a large number of natural hydrocarbon seeps and high petroleum-based vessel traffic (Horel et al., 2012), we hypothesized that the microbial communities of beach sands in this region may have adapted to this exposure. If so, the sand microbial communities from a beach without recent oil contamination and one with recent heavy oiling may have similar hydrocarbon degradation potential and would undergo similar community composition shifts following exposure to PAHs.

## Results and discussion

### Functional gene diversity

Berm sand from two different sites at St. George and Orange beaches collected at three different months after the DWH blow-out were subjected to non-expression functional gene microarray analysis. A total of 28,746 gene variants (between 19,150 and 23,337 persite and time, Table 1) representing 23.94% of the 120,054 unique sequences on the GeoChip 4.2 were detected. Functional gene variants are single sequence representatives of a functional gene with different sequences from diverse species or an overlapping sequence among species. The α-diversity, variation within-community, of functional gene variants was variable across the samples, but no significant differences in diversity indices were observed (*p* > 0.05, Table 1). This suggests that the presence of oil and its constituents had little effect on the overall functional gene diversity at the two beaches and over time. Collectively, 84% of detected gene variants were shared among the two different beaches and time points, suggesting a large core functional gene group is present among sands in this region of the Gulf of Mexico. Approximately 10% (2762) of all gene variants detected by the array were unique to Orange beach and 6% (1736) of the gene variants were unique to the non-impacted St. George beach, irrespective of the month of sampling. This represents a different resident functional gene community at each beach. Only 1–3% of all gene variants detected were unique to a specific beach and time (Table 1) representing a transient functional gene change. These differences in the detection of transient functional gene variants can be related to temporal variation, however, the greatest percentage (2.54%, Table 1) of unique gene variants were present during the detectable hydrocarbon sampling of June at Orange beach (Newton et al., 2013). The June sample at Orange beach had a hydrocarbon concentration 1000 times greater than any other sample and was the only sample collected when visible oil was washing ashore (Newton et al., 2013). Fluoranthene, phenanthrene, and pyrene were detected in the Orange beach sand in the month of June at 8.6, 89.4, and 5.3 μg kg^−1^, respectively. Unknown hydrocarbons with ten or more carbons in a chain (≥10C) were detected at 21.4 mg kg^−1^, as well.

**Table 1:**
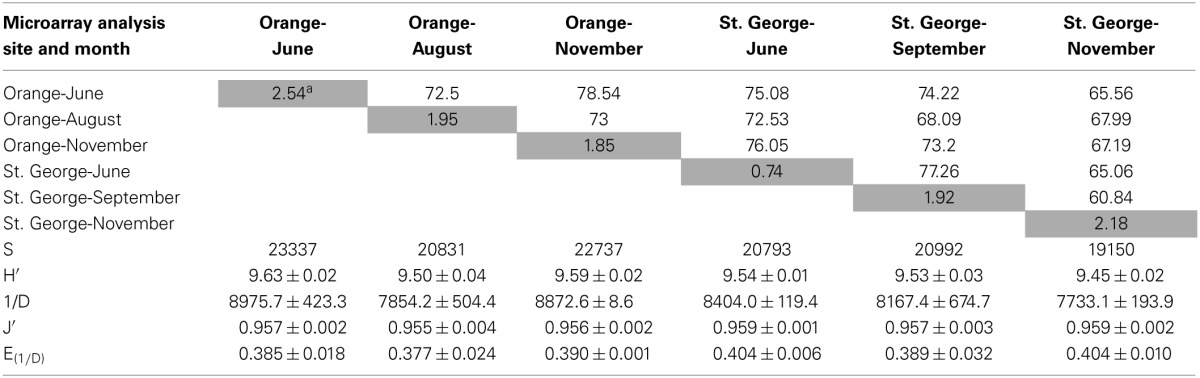
**Functional gene variant diversity, similarity, and evenness and percentage of unique and shared genes among samples**.

*S, Number of genes detected; H', Shannon-Weaver diversity index; 1/D, Inverse Simpson diversity index; J', Pielou (Shannon) evenness index; E_(1/D)_, Simpson evenness index*.

*^a^Numbers in shading indicate the percentage of gene variants detected at that site and time (S) that are unique*.

### Relative abundance of functional gene categories

**Figure 1:**
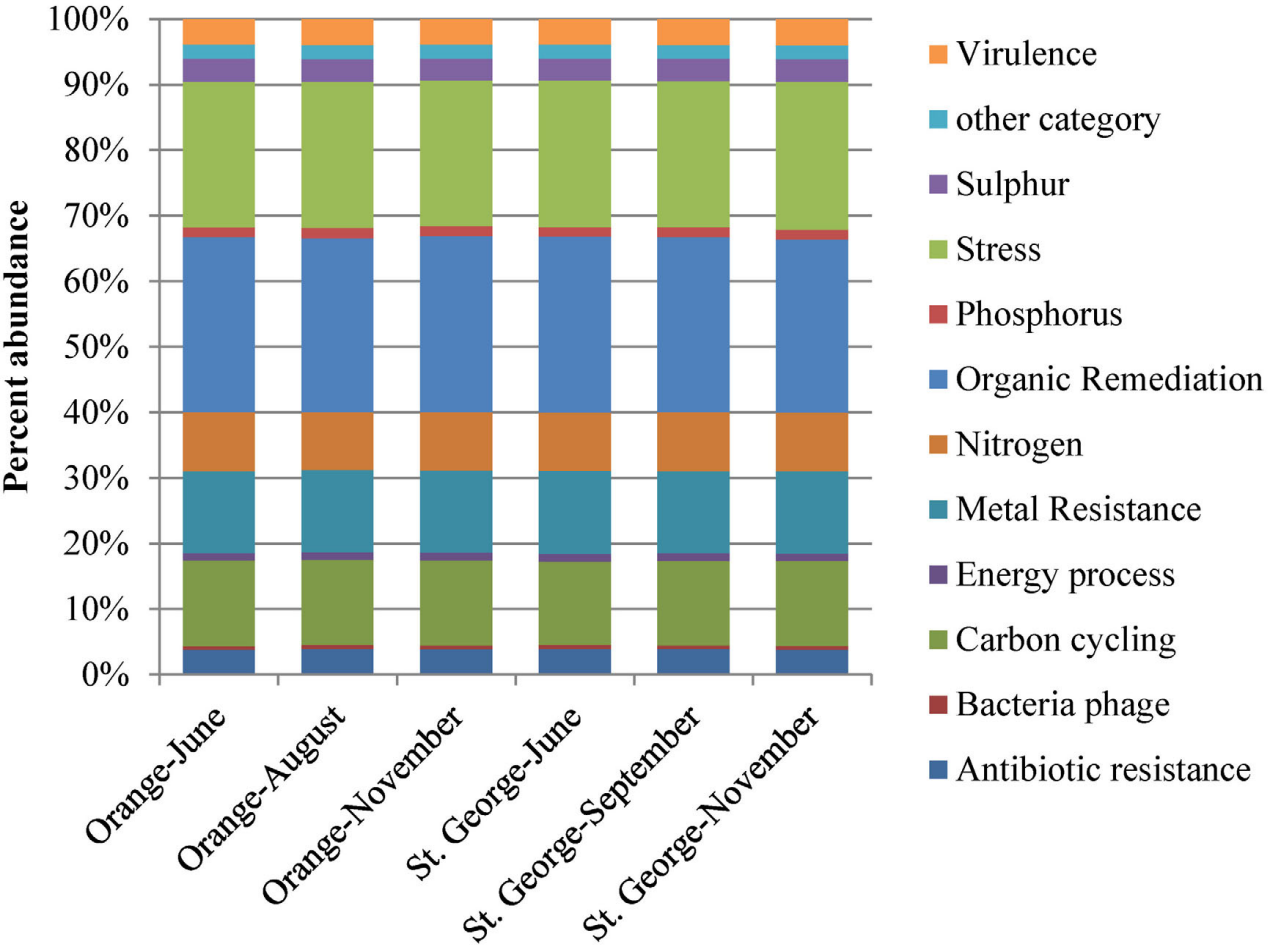
**Percent abundance of all functional gene groups detected by microarray analysis**. The total number of genes detected at each beach sands, St. George or Orange, was used to calculate the relative richness of each gene group. Values were averaged over the two samples from each beach.

The relative abundance of all functional gene categories was nearly identical across all sampling events at the two beaches and three time points (Figure 1). Overall, 27% of the detected gene variants were for genes involved in organic contaminants degradation; 13% in carbon metabolism; 12.5% in metal resistance; and 9% in nitrogen metabolism.

**Figure 2:**
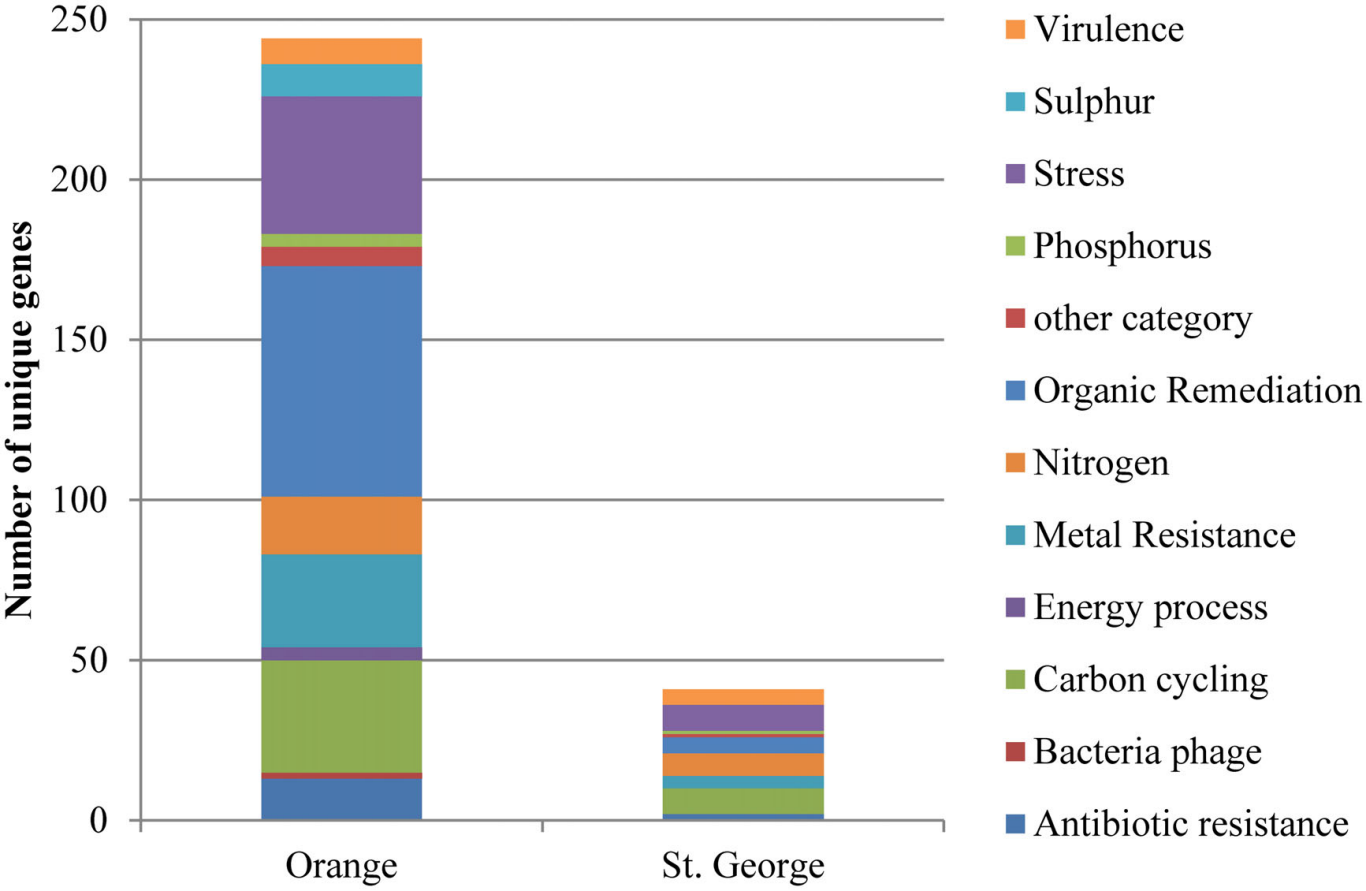
**Number of genes in functional gene groups unique to each beach detected by microarray analysis**. Genes were represented in the beach site when sands were collected at all three time points (June, August or September, and November 2010).

Of the unique gene variants detected of the residential functional genes (Figure 2) at Orange beach, 29% were involved in organic contaminant degradation, 7% in nitrogen metabolism, 14% in carbon metabolism, 12% in metal resistance, and 18% in survival of abiotic and environmental stress. The increased presence of these genes at the contaminated beach during and after the oiling event suggests that the oil and its constituents triggered small changes in the abundance of organisms carrying hydrocarbon and PAH degradation genes and genes involved in survival of abiotic and environmental stress. The unique gene variants detected were representatives of functional genes detected at both beach sites. The detection of more unique genes variants at Orange beach compared to St. George beach may be explained by the impact of the oil components on the intrinsic beach community and the addition of gene variants as the pelagic microbial community associated with the contaminated water reaches shore.

### Differences in the relative abundance of functional genes

The functional genes (sum of all gene variants detected) showed significantly greater (*p* < 0.05) relative abundance in the June sample at Orange beach than St. George beach (Table 2). These differences in abundance maybe directly related to the detection of fluoranthene, phenanthrene, pyrene and ≥10C hydrocarbons present at the Orange beach (Newton et al., 2013).

**Table 2:**
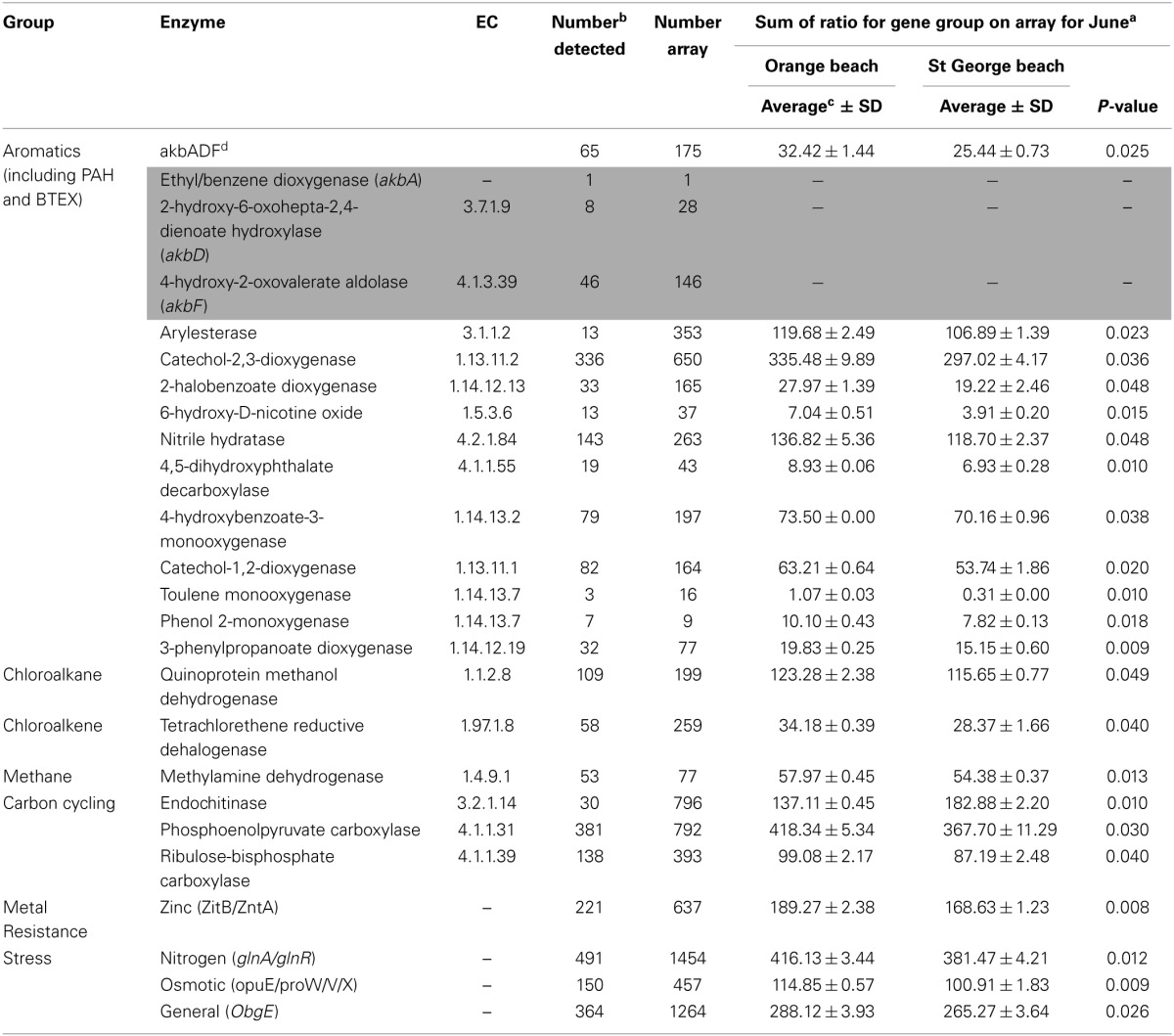
**Individual genes showing a significant increase (*p* < 0.05) at oil-impacted Orange beach compared to non-impacted St. George beach during the month of June**.

*^a^Average of the sum of the ratio between the gene variant spot fluorescence and average spot fluorescence on the microarray*.

*^b^Total number of gene variants detected (regardless of site) and used to generate the sum*.

*^c^Average of sum from two independent microarrays from samples 100 m apart at same beach*.

*^d^Average of the sum of the ratios of gene groups akbA, akbD, and akbF (shaded in gray)*.

Individual functional genes involved in the degradation pathways for benzoate, xylene, phthalate, toluene, chloroalkane and chloroalkene [Figure S1, KEGG pathway database (Kanehisa and Goto, 2000)] were greater in relative abundance in the Orange beach June sample. The majority of differentially detected functional genes in this sample were related to degradation pathways of aromatic hydrocarbons, PAH, and BTEX (benzene, toluene, ethylbenzene, and xylenes) suggesting increased concentration of these compounds following beach oiling. Aromatic hydrocarbons were also detected at Orange beach in the month of June (Newton et al., 2013). Additionally, genes involved in the chloroalkane and chloroalkene pathway were in greater abundance at Orange beach, suggesting the presence and possible accumulation of chlorinated hydrocarbons, feasibly from the degradation of chlorinated aromatics. Methylamine dehydrogenase, which forms formaldehyde from methylamine, was also abundant, indicating the possible accumulation of this single carbon amine from the degradation of more complex hydrocarbons and amino-aromatic compounds. Lu et al. (2012) showed significant increases in genes involved in initial oxidation of hydrocarbons such as *alkB* encoded alkane 1-monooxygenase and the *bbs* gene involved in anaerobic toluene degradation in the oil plume compared to non-plume deep sea waters during the DWH blow-out. In contrast there was no significant difference detected for these genes in our beach sand samples (*p* > 0.05). The lack of significant difference in *alkB* is unexpected as the increase in relative abundance of *Alcanivorax* and other known oil degraders (Newton et al., 2013) would suggest an accompanying detectable increase in the *alkB* gene, in particular the specific probe designed from *Alcanivorax*. This may reflect possible dissimilarity in sequences that are not represented in the arrayed probes (Zhou and Thompson, 2002). Oligonucleotide based arrays are sensitive to base-pair mismatches and exhibit high hybridization specificity (Cook and Sayler, 2003). This may explain the lack in the extent of change demonstrated in the functional gene array compared to the published changes in phylogeny (Newton et al., 2013). The changes in relative abundance that was detected of functional genes within the community structure indicate shifts in the degradation pathways of microbial community to optimally utilize available hydrocarbons between the initial plume and the hydrocarbons and derivatives that reached the shore.

The increased abundance of genes involved in PAH related degradation pathways in the Orange beach sand community compared to St. George beach suggest an increase in the microbial community members that can take advantage of the greater abundance of hydrocarbons. While the Orange beach microbial community had a greater relative abundance of genes involved in hydrocarbon and PAH degradation, these genes were also present at the non-impacted beach. These results suggest that the native communities found at these beaches have an innate potential for degradation of hydrocarbons and PAHs released during an oil spill.

There was a statistically greater abundance of genes involved in response to nitrogen limitation stress and osmotic stress (Table 2) in the heavily oiled beach compared to the non-impacted beach. This indicates the community members responding to hydrocarbons may harbor increased abundance of genes involved in nitrogen limitation and osmotic stress responses. Macro-nutrient limitation including nitrogen is common in oil contaminated communities as the hydrocarbons are utilized causing a decreased organic nitrogen pool (Atlas and Bartha, 1972; Leahy and Colwell, 1990; Toccalino et al., 1993). However, the nitrogen metabolism functional gene group, including nitrogen assimilation and nitrogen fixation, did not show significant differences (*p* > 0.05) in abundance between the two studied beaches. Functional gene analysis of the DWH deep-sea oil plume observed increased abundance of genes involved in nitrogen assimilation pathways, which was partially accredited to increase of biomass in the water column (Hazen et al., 2010; Lu et al., 2012). However, our results suggest that the community members responding to increased hydrocarbons and PAHs at the beach sands are not nitrogen fixers, but instead dealt with limited nitrogen via other mechanisms.

The greater abundance of osmotic stress genes in Orange beach sand compared to St. George beach could be associated with the hydrocarbon degrading community, as aromatic compounds have a direct influence on the membrane lipid bilayer, increasing its fluidity and thereby causing an increase in membrane permeability (Sikkema et al., 1994, 1995).

The genes encoding zinc transporters (*zntA* and *zitB*, Table 2) induced in the presence of high metal concentrations showed a greater abundance in the oiled beach. Since *zntA* can be responsible for the transport of zinc, lead and cadmium (Liu et al., 2005), this indicates the oiled community is more suited for responses of high metal concentrations. These metals were detected in oil-impacted waters and salt marshes (Liu et al., 2012; Joung and Shiller, 2013). It has been suggested that these trace metals increase as lower molecular weight hydrocarbons are degraded (Liu et al., 2012).

Interestingly, the carbon assimilation genes encoding phosphoenolpyruvate carboxylase and RuBisCO were also greater in abundance in the oiled beach (Table 2) suggesting an increase in the presence of autotrophic genes. The increase in genes harbored in mixotrophic bacteria might be related to the sensitivity of RuBisCO to oxygen in the oxygen limited and carbon dioxide rich environment. The presence of oil on sediment surface can inhibit the penetration of dissolved oxygen into the sediment and thus inhibit metabolic activity of heterotrophic bacteria utilizing the abundant hydrocarbons (Liu et al., 2012). It is also possible that the mixotrophic bacteria are taking advantage of available hydrocarbons (Subashchandrabose et al., 2013) and thus increasing the abundance of carbon assimilation genes.

While the presence of particular functional genes can be associated with changes in the beach community structure due to oiling, the change in the community composition can be associated with the pelagic microbial community in the contaminated water with already established PAH and hydrocarbon degrading microorganisms as it reaches shore.

Our findings suggest that oil contamination lead to enrichment of bacteria harboring functional genes involved in hydrocarbon degradation and related stress responses within the impacted sand microbial community.

### Degradation of a PAH mixture of naphthalene, fluorene, and benzo[α]pyrene

**Figure 3:**
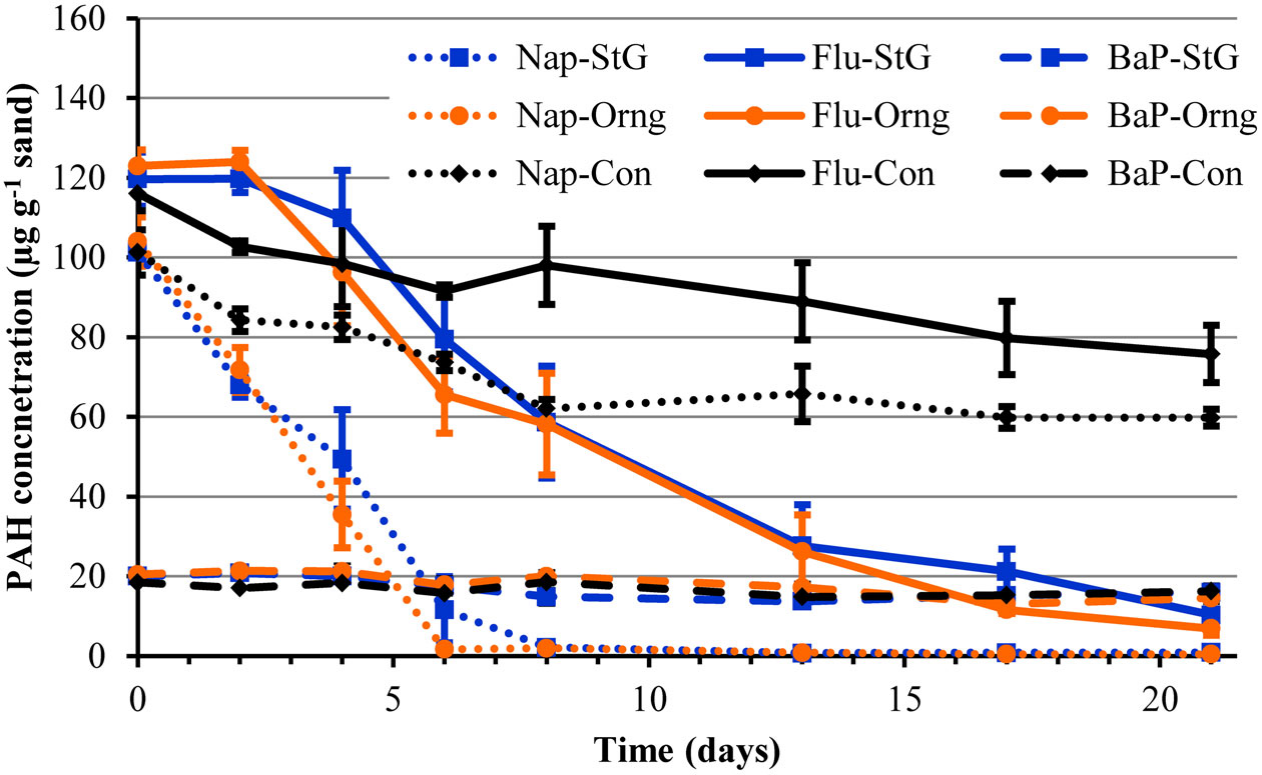
**Measured PAH concentrations in mesocosm columns containing sand from the oil impacted beach, Orange, and the non-impacted beach, St. George, over time**. The sand was spiked with a mixture of 100 μg g^−1^ naphthalene, 120 μg g^−1^ fluorene, and 20 μg g^−1^ benzo[α]pyrene prior to column setup. Lines in blue represent St. George (StG) sand mesocosm samples, lines in orange represent Orange (Orng) beach, and black lines represent control abiotic columns (Con). Solid lines represent fluorene (Flu) concentrations, dotted lines represent naphthalene (Nap) concentrations, and the dash line represents benzo[α]pyrene (BaP).

To further explore the intrinsic potential for PAHs degradation by the communities of Orange and St. George beaches, mesocosms utilizing sand from both locations were spiked with a mixture of PAHs. The PAH mixture contained naphthalene and fluorene, which were major PAH components of the source oil from the DWH blowout (Zhanfei et al., 2012), and benzo[α]pyrene a highly recalcitrant PAH that can also interfere with the biodegradation of other PAHs (Juhasz and Naidu, 2000). In the presence of the sand microbiome of Orange or St. George beach, naphthalene was quickly removed to undetectable levels by day 8, with significant (*p* < 0.05) concentrations removed by day 2 compared to abiotic degradation controls (Figure 3). There was no significant difference (*p* > 0.05) detected between the two beach microbial communities in their degradation potential. The maximum rate of depletion of naphthalene in Orange and St. George beach was 17.13 ± 6.88 and 14.67 ± 4.17 μg g^−1^ day^−1^, respectively, and was significantly greater (*p* < 0.05) than the abiotic disappearance rate of 3.77 ± 0.14 μg g^−1^ day^−1^. Measured fluorene concentrations did not show a significant difference (*p* > 0.05) to initial concentrations until day 6 indicating a lag in degradation by the microbial community or inhibition by the presence of initially higher concentrations of naphthalene (Figure 3). A significant difference (*p* < 0.05) in fluorene concentrations was observed by day 6 compared to the abiotic control columns. Orange and St. George beach communities were able to degrade fluorene with similar rates at 8.62 ± 1.29 and 10.57 ± 3.61 μg g^−1^ day^−1^, respectively. The rate of disappearance of fluorene in the abiotic control was 1.49 ± 0.14 μg g^−1^ day^−1^. Benzo[α]pyrene was not significantly (*p* > 0.05) degraded in any column during the duration of the measurements.

The results of the mesocosm experiment demonstrate the ability of the sand microbial community to quickly respond to an influx of PAHs such as those found in oil spills. The DWH blowout resulted in water partitioning of the more soluble PAHs during transport of the oil plume to the surface. This partitioning led to deep water plumes composed primarily of methyl-naphthalene and other two ring PAHs while the surface had a larger proportion of three ring PAHs such as fluorene (Diercks et al., 2010). Differential *in situ* degradation rates of PAHs also influence the composition of PAHs eventually impacting and accumulating along the shoreline. Sand collected at Pensacola Beach on September 1, 2010 showed the presence of benzo[α]pyrene and fluorene, while naphthalene was not detected (Kostka et al., 2011). At Orange beach in June, fluorene, naphthalene, and benzo[α]pyrene were not detected at the sampling sites while these PAHs were detected in other beaches such as those in Mississippi in June and August (Newton et al., 2013). We have shown that although fluorene and naphthalene haven't been detected at Orange and St. George beach in June, August, September, and November 2010 (Newton et al., 2013), sand microbial communities have the potential for the rapid degradation of these chemicals. A similar rapid response to crude oil and diesel fuel additions to mesocosms was found with uncontaminated sand from the Gulf of Mexico, suggesting microbial communities in this region adapt to hydrocarbons, including PAHs, as a result of exposure to natural oil seepages and high petroleum-based vessel traffic (Horel et al., 2012). While benzo[α]pyrene is not a common growth substrate for bacteria, experiments have shown that bacteria can degrade benzo[α]pyrene when grown on an alternative carbon source (as reviewed in Juhasz and Naidu, 2000). If the benzo[α]pyrene was bioavailable to the microbial community in the sand mesocosms, the available alternative carbon sources may not have been sufficient for co-metabolism of benzo[α]pyrene (Juhasz and Naidu, 2000). Alternatively, benzo[α]pyrene degrading bacterial species, more rare than low molecular weight PAH degraders, may not have been present in these sand communities (Haritash and Kaushik, 2009). However, this is unlikely due to the presence of known benzo[α]pyrene degraders such as *Sphingomonas, Mycobacterium, Pseudomonas*, and *Burkholderia;* and consortiums such as *Pseudomonas* with *Flavobacterium* (Juhasz and Naidu, 2000) within the mesocosm communities based on the 16S rRNA gene sequences described below. In summary, both beach communities were able to degrade lower molecular weight PAHs to the same extent, indicating the stored potential to degrade PAH contaminants is widespread in the beach sand environment along the Gulf Coast.

### Community composition in mesocosm column experiments

**Figure 4:**
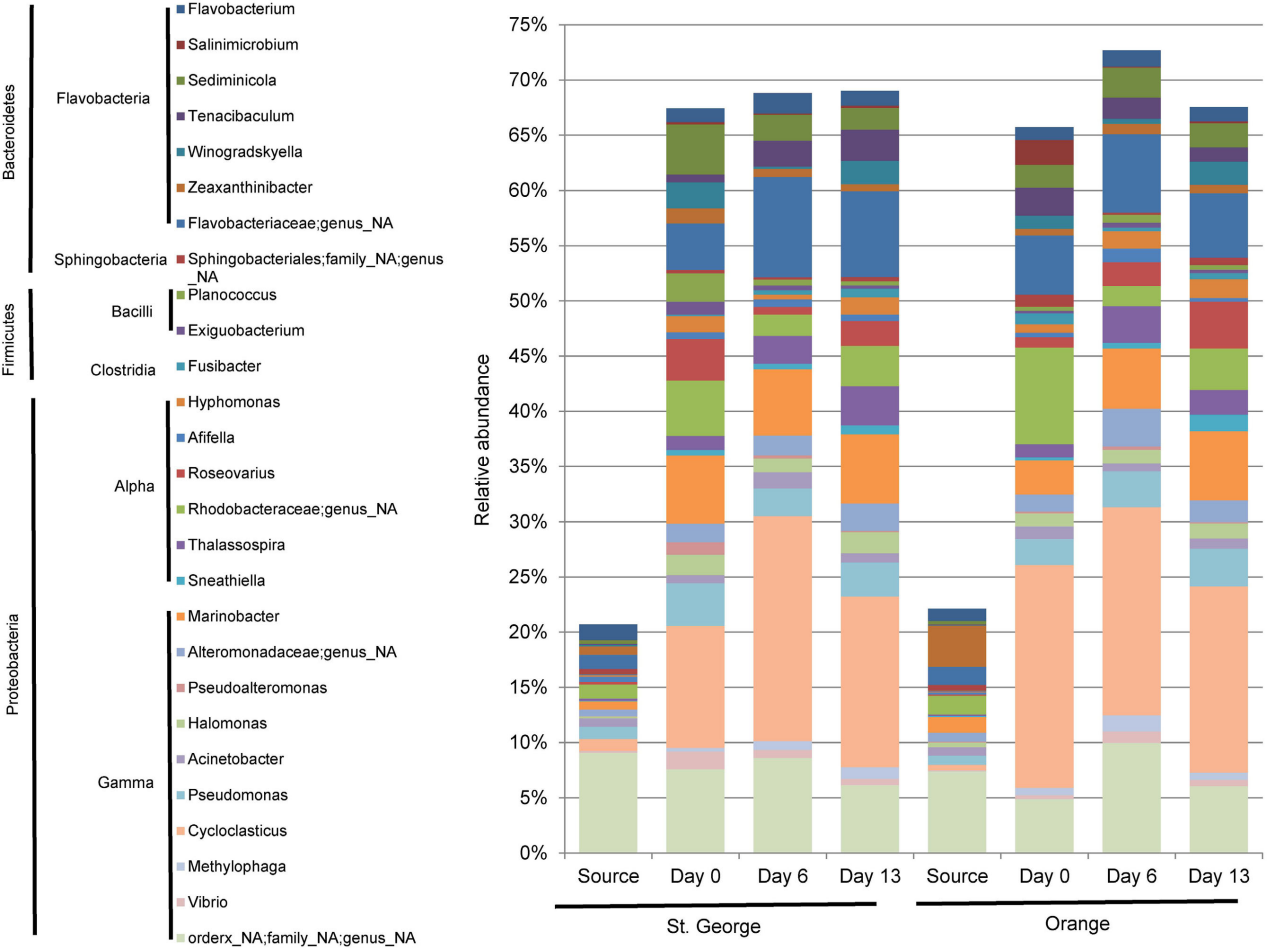
**Taxonomic representation of source and mesocosm column sand samples, assigned from the 16S rRNA gene sequencing data**. Genera (and un-resolved genera) composing ≥1% of the community at any time (day 0, 6, or 13) within any mesocosm (average of the 2 experimental columns) spiked with PAHs are represented. Source represents the population determined from the field sand sample, prior to manipulation of the sand for the mesocosm experiments. Day 0 represents approximately 6 h during the assembly of the mesocosm experiments in which the bulk of the sand samples were mixed with PAH containing sand mixture. A genus was considered only if it constituted ≥0.01% of the community from any single sample.

The abundance of bacterial 16S rRNA gene sequences revealed little difference between the two beach sand mesocosm microbial community compositions during the PAH depletion mesocosm experiments (Figure 4, Table S1). Sequencing of environmental DNA extracted from the sand mesocosm samples generated over 7.9 million high quality V6 region bacterial rRNA gene tags. Overall, 27 taxa (22 genera and 5 additional unresolved taxa) each constituting ≥1% of the bacterial community were found post acclimation at any time point (days 0, 6, or 13) in either the Orange or St. George mesocosms (averaged over the individual columns; Figure 4). These taxa represented ≥65% of the population within the individual mesocosm community. In contrast these taxa represented ≤25% of the population in the field sand samples (source) used to set up the mesocosms. Although the identity of the taxa in the field and mesocosm communities was similar there were dramatic change in the relative abundances of taxa between the post acclimation, day 0 and the field sample (Source; Figure 4). These changes are likely due to positive and negative selection of the taxa during the 6 h time frame of mixing and evaporation of the acetone used to apply the PAHs during the initial assembly of the mesocosms at room temperature. The PAHs spiked within the mesocosms did not show detectable depletion when the sand was sampled after acclimation on day 0 (Figure 3).

The core community representing ≥1% of the community in both mesocosm in all three time points (included in Figure 4) were identified in the mesocosm samples included representatives of *Flavobacteria*: *Flavobacterium* and *Sediminicola; Alphaproteobacteria: Thalassospira; and Gammaproteobacteria: Cycloclasticus*, *Marinobacter*, *Halomonas, and Pseudomonas*. With the exception of *Sediminicola*, these genera were reported to be highly associated with oil degradation during the DWH incident (Kostka et al., 2011; Chakraborty et al., 2012; Kimes et al., 2013; Liu and Liu, 2013; Newton et al., 2013). *Sediminicola* was reported as a major member of the microbial community found in the chronically oil and PAH contaminated Liaodong Bay of Bohai Sea, China (Zheng et al., 2014). *Thalassospira* is a known PAH-degrader of fluorene and naphthalene (Kodama et al., 2008) and was associated with oil on the water surface during the DWH blowout (Liu and Liu, 2013). *Cycloclasticus* represented between 15and 20% of the community in the columns, while only 1.1 and 0.5% in the St. George and Orange beach sands used for the mesocosms, respectively. A similar rapid increase in *Cycloclasticus* under experimental laboratory conditions have been reported previously (Kasai et al., 2002). Studies in marine environments have described *Cycloclasticus* as a primary contributor to PAH degradation (Kasai et al., 2002; Wang et al., 2008; Jurelevicius et al., 2013) during oil spills (Hazen et al., 2010; Chakraborty et al., 2012; Mason et al., 2012). *Marinobacter* is also well-established in alkane and PAH degradation (Vila et al., 2010). Newton at al. reported also that in the one sample set collected while oil was visibly washing ashore (Orange Beach June) OTUs associated with *Marinobacter* were largely increased in the community. *Pseudomonas* is able to degrade naphthalene in experimental systems and is thought to be involved in the co-metabolization of fluorene (Stringfellow and Aitken, 1995). *Flavobacterium* have been associated with PAH degradation (Trzesicka-Mlynarz and Ward, 1995). Within a mixed culture of *Flavobacterium* sp. and *Pseudomonas* sp., the degradation of benzo[α]pyrene was possible (Trzesicka-Mlynarz and Ward, 1995). Despite the enrichment of both, *Flavobacterium* and *Pseudomonas* sp. in our mesocosms, benzo[α]pyrene was not degraded in any column during the duration of the measurements suggesting it wasn't bioavailable or longer time is needed for the microorganisms to deplete this pollutant. *Sediminicola* has been associated with oil and PAH contaminated waters (Zheng et al., 2014). The large sequence representation of this core community of known and associated PAH degraders in the mesocosms suggests that biodegradation potential for hydrocarbons, including PAHs, was quickly established in the sand microbial communities prior to detectable degradation of the spiked PAHs.

**Figure 5:**
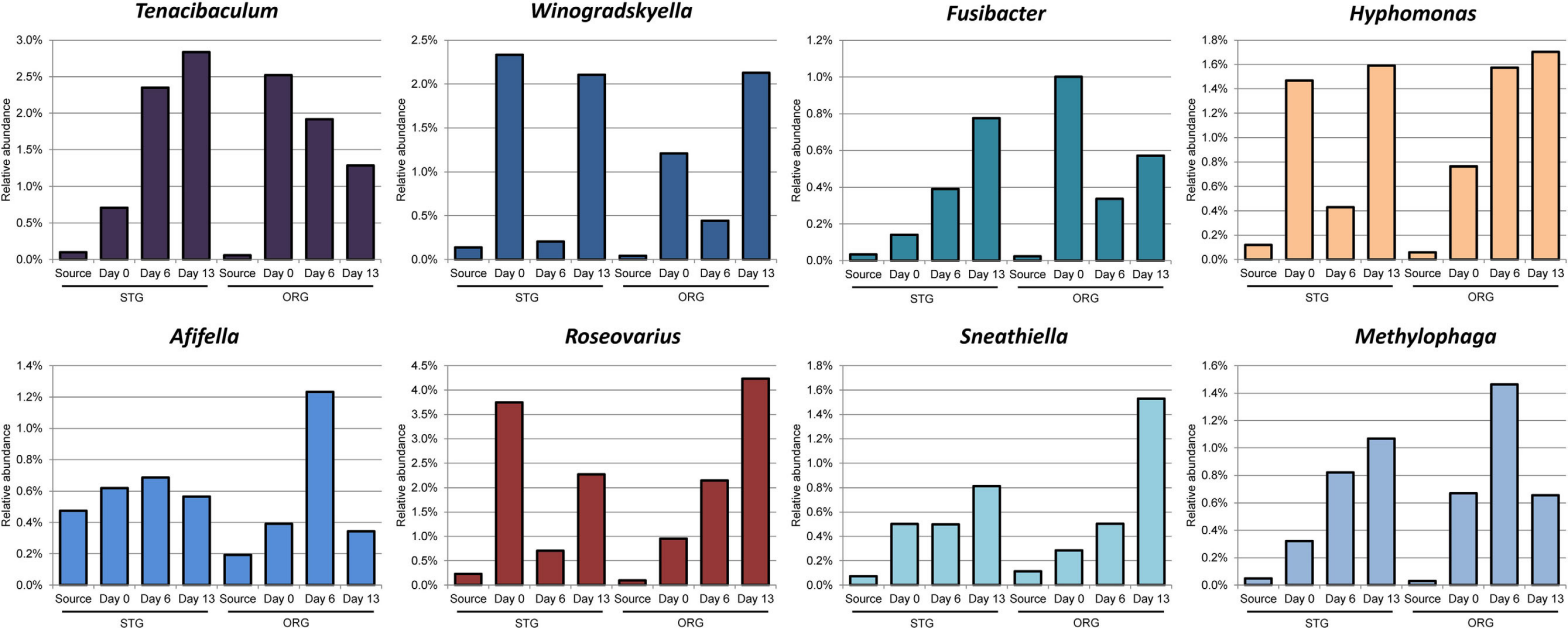
**Bacterial genera enriched within the microbial community in the mesocosm columns**. Only genera demonstrating a ≥3-fold increase in relative abundance when compared between any two time points (Days 0, 6, and 13) of at least one sand microbial community (average of 2 replicate mesocosm columns for each beach sand source) and represented in Figure 4. Source represents the population determined from the field sand sample, prior to manipulation of the sand for the mesocosm experiments. Day 0 represents approximately 6 h during the assembly of the mesocosm experiment in which the bulk of the sand samples were mixed with PAH containing sand mixture. Bar graphs in color are for genera abundant above 1% in the community, depicted with the same color as in Figure 4.

Eight genera of the community presented in Figure 4 exhibiting a ≥3 fold increase in average community percentage between any two time points post acclimation (Figure 5) denote a rapidly responsive community. The genera *Afifella, Methylophaga, Winogradskyella, Hyphomonas, Roseovarius, Tenacibaculum, Fusibacter*, and *Sneathiella* showed dynamic changes in relative abundance that may be related to the role they play in their respective communities involved in the degradation of naphthalene and fluorene.

*Tenacibaculum* is known to produce bacteriolytic enzymes allowing nutrient recycling, which may account for their increase in abundance (Banning et al., 2010; Dubinsky et al., 2013). *Tenacibaculum* is as yet not associated with direct PAH utilization. *Winogradskyella* was among the core community present in the Gulf of Mexico beach sand following the period of beach oiling from the DWH blowout (Newton et al., 2013) and has been enriched previously with petroleum in microcosms (Zhao et al., 2014). *Fusibacter* species have been associated with oil producing wells (Ravot et al., 1999) and contaminated sites containing chlorinated solvents (Lee et al., 2011). *Fusibacter* prefers anaerobic environments and as yet been determined to be involved in direct degradation of PAHs (Ravot et al., 1999; Lee et al., 2011). *Hyphomonas* abundance has been associated with crude oil added to natural sea-water in microcosms (Coulon et al., 2007). *Hyphomonas* has also been associated with degradation potential of hydrocarbon-contaminated sediments from a harbor in the Tirrenean Sea after the addition of sources of nitrogen and phosphate (Yakimov et al., 2005). *Roseovarius* was one of the genera of numerous Alphaproteobacteria detected in sequences associated with a sample 127 km from the DWH rig (Kimes et al., 2013). *Roseovarius* is also known to be degraders of low molecular weight PAHs (Gallego et al., 2013). *Afifella* is a photosynthetic purple, non-sulfur bacteria that has not be associated with hydrocarbon or PAH degradation (Urdiain et al., 2008). *Methylophaga* have been associated with the later phases of the DWH oil spill and has been suggested to be active in the heterotrophic community that was consuming residual cell mass and organic residues (Dubinsky et al., 2013). *Sneathiella* is a halotolerant, aerobic, chemoheterotroph that has not yet been associated with PAH or oil degradation activities (Jordan et al., 2007). Since a number of these genera have been associated with PAH and hydrocarbon degradation communities, their presence may be necessary to support the PAH depletion within the mesocosms and directly impact the ability of other genera to degrade PAHs within the columns.

The presence of the core community with known and associated PAH degraders in all mesocosm columns during the sampling times and their presence during the DHW spill may point to members of these genera as key players in the PAH degradation by the coastal sand microbial community during hydrocarbon degradation. The responsive community which contained associated and yet unassociated PAH degraders in the mesocosm columns may indicate a different involvement of these genera in the degradation of PAHs within the sand communities with different oiling history.

## Conclusions

Our results indicate that a variety of genes related to hydrocarbons degradation and stress were enriched in response to the oil contamination and associated environmental changes. Specifically, we observed enrichment of metabolic genes involved in the degradation pathways for BTEX, alkanes, alkenes and aromatic hydrocarbons including PAHs. In addition, genes involved in metabolic processes related to osmotic stress and metal transport showed relative increases most likely related to the oil contamination. However, the relative abundance of the majority of functional gene categories investigated was similar across the two sampled beaches and 5-months separating sampling time points. These results suggest that sand microbial communities are relatively functionally stable and that the hydrocarbon exposure adds a layer of complexity to the system without initiating drastic community change.

The potential of microbial PAH degradation between beaches with a different history of oil contamination was tested in a laboratory column experiment. Upon exposure with a PAH mixture we observed similar biodegradation potential from each beach sand community for the mixture despite of its oiling history. The lower molecular weight PAHs naphthalene and fluorene were depleted with similar rates, while the concentration of benzo[α]pyrene remained constant in all mesocosms for the time of incubation. Further study using nutrients and electron donors or acceptors additions is needed to understand if these communities are also primed to degrade recalcitrant pollutants such as benzo[α]pyrene and other higher molecular weight PAHs.

The community composition of the PAH degradation in the mesocosms consisted of a core community of known or associated PAH degraders that was consistent among the two sand communities. A responsive community to the PAH addition that showed different shifts in abundance between the two sand mesocosms and may be related to the availability of specific PAHs was also detected. The observed similarity in the ability of the two differently oil impacted microbial communities to degrade PAHs and the presence of a core community containing PAH degraders indicate the wide spread dissemination of PAH degraders on beaches over large distances. This is most likely explained by the large number of natural hydrocarbon seeps and high petroleum-based vessel traffic in this region (Horel et al., 2012) which may select for a PAH and hydrocarbon consortium within the microbial community of the pelagic and coastal waters, as well as, distant beaches these consortiums will eventually reach.

Further study is needed to understand the response of microbial communities not only to change in oil pollutant concentrations but also to stressors such as nutrient starvation, anoxia, and ecological interactions in the Gulf of Mexico coastal sand ecosystems. Experimental mesocosms with stratified conditions to explore the impact of oxygen, electron acceptors, nutrients, and oil pollutant micro-concentration gradients will be particularly useful to address ecological questions under controlled conditions. Combined with meta-omics' technologies this approach provides powerful tools to gain insights into functional networks involved in hydrocarbon degradation in coastal sandy sediments. A global understanding of the impact of oil on indigenous coastal microbial community structures, function, and resilience will help in development of strategies for restoring ecological balance following oil spills in coastal areas.

## Materials and methods

### Study sites and sampling procedures

For functional gene microarray analysis, surface sand samples were collected from two beaches in the southeast United States along the Gulf Coast in Alabama, and Florida as described in (Newton et al., 2013). The beach locations referred to in this study are: Orange beach (Cotton Bayou Beach area in Alabama, 30° 16' 54” N, 87° 41' 17” W), and St. George (St. George Island, Florida, 29° 41' 22” N, 84° 46' 59” W). Exposed surface sand (wet intertidal sand located at the high point of wave action on the beach face) was sampled at each beach. For the sand environments, we chose two sampling sites ~100 m apart at each beach location. Samples were collected during five sampling periods: June 13–15, 2010, August 8, 2010, September 20–22, 2010, November 15–18, 2010, and August 15–17, 2011, except for St. George, which we were unable to collect during the August 2010 period. Following collection in the field, the sand samples were stored on ice between 2 and 28 h during the sampling expedition and then on dry ice before being shipped to the lab for further processing. In the lab, sand was stored at −80°C prior to DNA extraction procedures. Sand for the mesocosm experiments was sampled from Orange beach and St. George in excess of 4 kg during a sampling trip in August 2011 and were stored on ice and then stored at 4°C until use in the mesocosms.

Based on reports by NOAA's cleanup and assessment techniques, Orange beach was classified as heavily oiled following the DWH spill, while St. George had either trace or no oiling and was referred to as a control or un-impacted site in their assessment and in a previous study (Newton et al., 2013). St. George also serves as the no recent oil contamination control beach for all analyses in this study.

### DNA extraction

DNA extractions were performed on 5 or 1 g of sand samples essentially as Zhou et al. (1996) described but without CTAB and reduced volumes to scale. Rather than using chlorophorm: isoamyl alcohol, the samples was purified by selective RNA precipitation using 1.5 M ammonium acetate and the Wizard Genomic DNA purification kit (Promega) for protein precipitation, DNA precipitation and DNA rehydration steps. DNA was quantified and checked for quality by NanoDrop spectrophotometry and with 1% agarose gel electrophoresis.

### Microbial community DNA labeling, microarray hybridization and analysis

For microarray analysis, 1.5 μg of DNA extracted from 5 g sand sediment was labeled with Cy3 fluorescent dye (GE Healthcare, Piscataway, NJ, USA) by random priming (Wu et al., 2006; Van Nostrand et al., 2009). The labeled DNA was purified, hybridized to GeoChip 4.2, and processed as described in Lu et al. (2012). GeoChip 4.2 is a functional gene array (Hazen et al., 2010; Lu et al., 2012) containing 120,054 distinct probes and covers 200,393 coding sequences in different microbial functional and biogeochemical processes. Spots with signal-to-noise ratios lower than 2 were removed before statistical analysis was performed (He et al., 2010).

All GeoChip hybridization data are available at the Institute for Environmental Genomics, University of Oklahoma (http://ieg.ou.edu/). The data was pre-processed to response ratios of each gene variant, which is the ratio of intensity of the variant to the average of all genes on the array. Simpson's, Shannon-Weiner's, and evenness indices were calculated to access functional gene diversity. ANOVA was used to determine significant differences between the functional microbial communities between the two beach sites and samplings over time. Tukey's test was used for pairwise comparisons. A significance level of *p* < 0.05 was used for all comparisons (He and Wang, 2011). Total abundance of each gene category or all gene variants representing a functional gene were calculated as the sum of response ratio and compared between the beach locations overtime using ANOVA and Tukey's test for follow-up pairwise comparison.

### PAH degradation potential of the sand microbial community

The potential for degradation of PAHs was tested in eight constructed mesocosms, three replicates with native sand communities collected August 2011 for each beach, Orange and St. George, and two abiotic controls. After sampling in the field, the sand was shipped on ice and a stored at 4°C, until used in the mesocosm construction. Glass columns (Chemglass; 7.62 cm inner diameter by 30.5 cm effective length), fitted with fritted glass supports, were filled with sand from Orange beach and St. George beach spiked with a mixture of three low molecular weight PAHs, naphthalene, fluorene, and benzo[α]pyrene. Stock concentrations of fluorene, naphthalene, and benzo[α]pyrene in acetone was hand mixed into 350 g batches of sand from St. George or Orange beach for 1 min to achieve final concentrations of 100 μg g^−1^ naphthalene, 120 μg g^−1^ fluorene, and 20 μg g^−1^ benzo[α]pyrene in the columns. The acetone from PAH mixed sand was allowed to evaporate for 1.5 h at room temperature prior to assembly of the columns. A total of 1.4 kg of sand mixed with PAHs was used to fill each of three glass column replicates for each beach. The time 0 samples were taken approximately 6 h after the initial spike of the first batch of sand which is referred to as the acclimation period. To setup two abiotic control columns, sand from St. George beach was autoclaved to remove the live microbiome prior to addition of PAH as for the other columns. Sodium azide was added to the artificial sea water for the control columns at 0.1%. To all columns, 400 mL of sterile artificial sea water (Kester et al., 1967; Berges et al., 2001) was added and allowed to drain by gravity flow and captured. Filling and draining (400 ml volume) with artificial seawater occurred over 3-h periods. The mesocosms were left filled and drained for 3 h each recreating a 12-h tidal period. The drained sea water was re-circulated to the top of the column by peristaltic pump. Viton tubing (0.32 cm o.d.) was used for all applications that involved contact with effluent water. The columns were operated over a 21 day period at room temperature (25 ± 3°C).

Sand was sampled by sterile metal tubing core from each glass columns at the time points indicated in Figure 4 up to 21 days. For extraction of PAH, a recovery standard of 200 μg phenanthrene-d10 to 10 g of sand sample was used and showed a recovery of 95%. The sand was extracted three times with a total of 10 mL n-hexane. Samples were stored at 4°C until analysis and analyzed within 2 days. PAHs and phenanthrene-d10 was quantified by gas chromatograph analysis using Agilent model 7890A, equipped with flame ionization detector. The GC column temperature was 55–250°C, programmed at 3°C/min to 75°C hold for 3 min; 5°C min^−1^ to 100°C; and 20°C min^−1^ to 250°C hold for 60 min. Helium was used as a carrier gas, at a pressure 25 psi. For detector, the flow of hydrogen, air and nitrogen were 30, 400 and 25 mL min^−1^, respectively. Injector and detector temperatures were 250 and 300°C, respectively.

### Bacterial 16S ribosomal gene tag sequencing

We amplified bacterial 16S rRNA gene V6 hypervariable regions in our samples according to protocols developed at the Josephine Bay Paul Center at the Marine Biological Laboratory, Woods Hole, MA (Eren et al., 2013; Morrison et al., 2013). For this project sequencing was carried out on 12 mesocosms samples: one each from four mesocosms at three time points: Day 0 after hydrocarbon conditioning (~6 h post-addition), Day 6 and 13. Two mesocosms contained sand from St. George and two contained sand from Orange beach. Once obtained, sequence reads were quality-filtered according to Eren et al. (2013). Additionally, three samples (two sites at St. George and one site at Orange beach) from August 2011, collected at the same location and time as the mesocosm source samples, were sequenced according to the procedures described above. Taxonomy was assigned using GAST (Huse et al., 2008) and the data was uploaded to the Visualization and Analysis of Microbial Population Structures website (VAMPS: http://vamps.mbl.edu). The amplification, sequencing, and processing of all 16S rRNA gene community sequence data derived directly from beach sand samples in 2010 is described in (Newton et al., 2013). All mesocosm sequence files described in this study are available at the National Center for Biotechnology Information (NCBI) Sequence Read Archive, Bioproject (PRJNA244096) and for the previously published beach samples (Newton et al., 2013) under Bioproject PRJNA208242. All analyses were performed on averages of the duplicate mesocosms at each time point.

### Conflict of interest statement

The authors declare that the research was conducted in the absence of any commercial or financial relationships that could be construed as a potential conflict of interest.

## Supplementary material

Table S1 **16S rRNA gene sequence taxonomical diversity, similarity, and evenness in mesocosms**.

Figure S1 **Diagram illustrating the inclusive metabolic pathways (based on KEGG Pathway Database) representing the genes involved in PAH and hydrocarbon degradation**. The genes were determined by functional microarray analysis that showed greater abundance in the month of June at Orange beach (heavily oiled) compared to St. George beach (non-impacted) sands. The genes are listed in the same order as in Table 2.
