# Supplementary material for: The polycyclic aromatic hydrocarbon degradation potential of Gulf of Mexico native coastal microbial communities after the Deepwater Horizon oil spill
Source: Front Microbiol. 2014 May 9;5:205. doi: 10.3389/fmicb.2014.00205 (PMC4023046; doi:10.3389/fmicb.2014.00205)
Supplement: Table S1 — 16S rRNA gene sequence taxonomical diversity, similarity, and evenness in mesocosms. [file Presentation1.PDF]

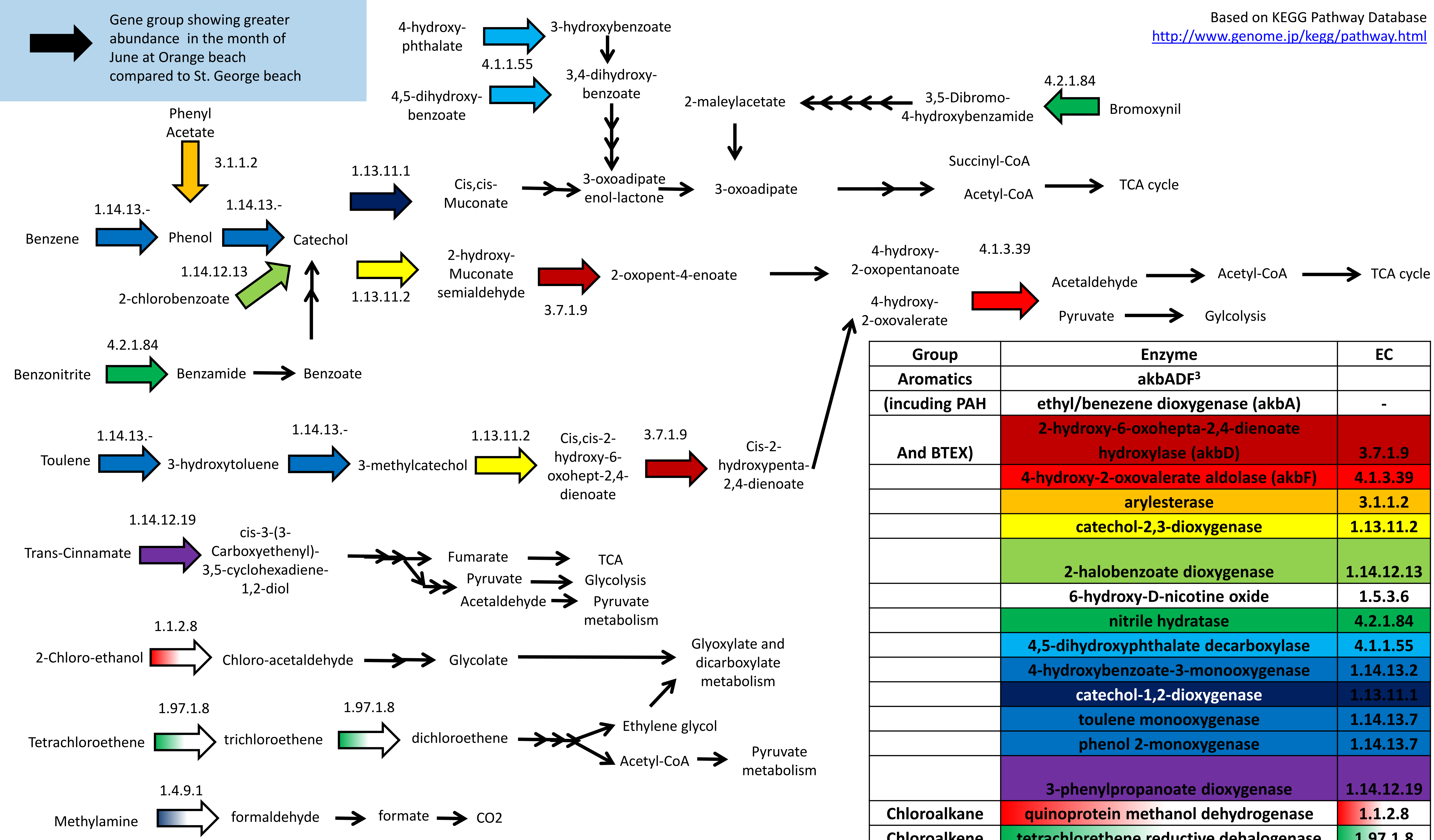

Group	Enzyme	EC
Aromatics	akbADF[3]	
(incuding PAH	ethyl/benezene dioxygenase (akbA)	-
And BTEX)	2-hydroxy-6-oxohepta-2,4-dienoate hydroxylase (akbD)	3.7.1.9
	4-hydroxy-2-oxovalerate aldolase (akbF)	4.1.3.39
	arylesterase	3.1.1.2
	catechol-2,3-dioxygenase	1.13.11.2
	2-halobenzoate dioxygenase	1.14.12.13
	6-hydroxy-D-nicotine oxide	1.5.3.6
	nitrile hydratase	4.2.1.84
	4,5-dihydroxyphthalate decarboxylase	4.1.1.55
	4-hydroxybenzoate-3-monooxygenase	1.14.13.2
	catechol-1,2-dioxygenase	1.13.11.1
	toulene monooxygenase	1.14.13.7
	phenol 2-monooxygenase	1.14.13.7
	3-phenylpropanoate dioxygenase	1.14.12.19
Chloroalkane	quinoprotein methanol dehydrogenase	1.1.2.8
Chloroalkene	tetrachlorethene reductive dehalogenase	1.97.1.8
Methane	methylamine dehydrogenase	1.4.9.1

Table S1. Mesocosm 16 S sequence taxonomical diversity, similarity, and evenness

Mesocosm Sand Location and Day of Sampling	Sample Depth	Observed Richness	ACE	Chao1	Shannon-Weaver Diversity (H’)	Simpson Diversity (D)	Inverse Simpson (1/D)
St. George Source	627452	42.5	42.6	42.6	1.55	0.67	3.01
St. George Day 0	596667	40.0	40.3	40.5	1.23	0.63	2.69
St. George Day 6	588054	40.0	40.5	40.6	1.18	0.62	2.64
St. George Day 13	672613	39.0	39.5	39.8	1.22	0.63	2.69
Orange Source	418499	41.8	41.8	41.8	1.54	0.67	3.03
Orange Day 0	541670	40.0	41.1	42.6	1.21	0.63	2.68
Orange Day 6	607403	40.0	40.8	44.0	1.15	0.61	2.58
Orange Day 13	672613	39.0	39.5	39.8	1.22	0.63	2.69
